# Resilience of auxiliary nurses providing nursing care to patients with intellectual disabilities at a public mental healthcare institution

**DOI:** 10.4102/curationis.v42i1.1954

**Published:** 2019-08-20

**Authors:** Steven D.M. Nthekang, Emmerentia du Plessis

**Affiliations:** 1 Witrand Psychiatric Hospital, Potchefstroom, South Africa; 2 School of Nursing Science, North-West University, Potchefstroom, South Africa

**Keywords:** resilience, care delivery, auxiliary nurses, intellectual disability, practical wisdom

## Abstract

**Background:**

Although mental health is regarded by the International Council of Nurses as a very important element of wellness, healthcare to patients with intellectual disabilities still remains neglected and under-resourced in most societies. Auxiliary nurses are crucial in providing nursing care to patients with intellectual disabilities. These nurses may not be prepared to handle challenges in providing nursing care to these patients, but their resilience can help them to manage these challenges. Limited research is available with regard to the resilience of auxiliary nurses providing nursing care to patients with intellectual disabilities.

**Objectives:**

To explore and describe the perceptions of auxiliary nurses providing nursing care to patients with intellectual disabilities on their resilience and protective mechanisms and vulnerability factors that influence their resilience when providing nursing care to these patients.

**Method:**

A qualitative, descriptive inquiry approach was used. The population comprised approximately 220 auxiliary nurses providing nursing care to patients with intellectual disabilities at a mental healthcare institution. Auxiliary nurses were selected through purposive sampling with the assistance of a mediator. The sample size was determined by data saturation. The data were collected through four focus group interviews with altogether 32 participants.

**Results:**

Five main themes emerged from the data. Practical wisdom was applied by the participants. They also made use of different forms of interactions, including the application of strategies such as utilising induction programmes and being willing to learn, in order to remain resilient. Protective mechanisms and vulnerability factors influence their resilience.

**Conclusions:**

Recommendations to strengthen the resilience of auxiliary nurses caring for patients with intellectual disabilities were formulated from the research findings, including recommendations for nursing practice, education and nursing research. Informal peer support, as well as addressing ethical issues, improving nurse–patient communication, training to handle adverse working conditions, and continuing education and further research on the practical wisdom of auxiliary nurses, is recommended.

## Introduction and background

Patients with intellectual disabilities are prone to specific health needs and common behavioural problems, such as self-injury and aggression (Hensel, Lunsky & Dewa 2012:910). Research shows that caring for patients with intellectual disabilities may pose challenges, and healthcare professionals are, therefore, put under considerable strain (Hensel et al. 2012:910). Amidst these difficult circumstances, auxiliary nurses continue to provide mental healthcare to all patients − including patients with intellectual disabilities (Stubbs & Dickens 2008:1).

Healthcare professionals working with patients who are intellectually disabled report intense emotions because of the aggressive and challenging behaviour of these patients (Storey, Collis & Clegg 2011:235). Violent acts from these patients make auxiliary nurses feel vulnerable, and if they are not adequately trained in how to handle violence at their workplace, continual exposure to violence can have a negative impact on their work commitment (Camerino et al. 2008:48; Gillespie et al. 2010:178). It is also important to mention that to counteract these acts of violence and aggression, some auxiliary nurses maintain a no-tolerance policy as a protective mechanism to reduce the risk of physical harm because of aggressive behaviour (Gillespie et al. 2010:181).

Although they are considered semi-skilled labourers and despite the fact that they are classified as the lowest category of nursing staff, auxiliary nurses perform complex jobs accompanied with serious responsibilities (Cherry, Ashcraft & Owen 2007:183). Considering that patients who are intellectually disabled can exhibit aggressive behaviour, auxiliary nurses are particularly at risk of bearing the brunt of the aggressive and violent behaviour of these patients, because they often operate on the ‘front line’ of care (Bernstein & Saladino 2007:301).

It is, therefore, important that nurses – auxiliary nurses included – who provide nursing care develop resilience to adjust successfully to the demanding physical, mental and emotional nature of the mental healthcare environment (Cameron & Brownie 2010:66). Developing the ability to thrive in an unpredictable healthcare environment is crucial for auxiliary nurses (Coyne 2008:3157). These nurses work with individuals whose daily lives are characterised by hardships, and resiliency is therefore urgently needed as a coping mechanism (McGee 2006:45).

This article reports on research regarding the resilience of auxiliary nurses working at a public mental healthcare institution.

## Problem statement

While auxiliary nurses are performing their caring duties, they can be exposed to the aggressive behaviour of patients with intellectual disabilities. According to Shields and Wilkins (2009), there is evidence that suggests that auxiliary nurses experience disproportionate levels of patient aggression when compared to other healthcare workers. The training of auxiliary nurses does not prepare them for the spectrum of problems they encounter while performing their duties (Stone & Dawson 2008:52).

It is evident that their training does not prepare them to be resilient to deal with these kinds of difficulties, and they are, therefore, put at risk of applying ineffective coping mechanisms and exhibiting inappropriate behaviour in response to the aggressive and challenging behaviour of patients with intellectual disabilities.

They, therefore, need resilience as a coping mechanism to be able to effectively care for patients with intellectual disabilities (Edward 2005:142–143). However, at the onset of this research very limited information was available on the perceptions of auxiliary nurses caring for patients with intellectual disabilities concerning their resilience. This paucity of information was identified as a problem in this study, because such information can guide the facilitation of the resilience of auxiliary nurses.

### Purpose of the study

The purpose of this study was to explore and describe the perceptions of auxiliary nurses caring for patients with intellectual disabilities on their resilience, and their perceptions on protective and vulnerability factors that play a role in their resilience when caring for patients with intellectual disabilities.

### Definitions of key concepts

*Resilience*: The ability of individuals to adjust to unfavourable conditions in a positive way (Jackson, Firtko & Edenborough 2007:1) and to bounce back from hardships and to overcome negative life experiences (Greeff & Ritman 2005:38), such as aggressive and violent behaviour of patients with intellectual disabilities.

*Auxiliary nurses*: Individuals who are trained to provide elementary nursing care, according to the prescribed level (South African Nursing Council 2005). In this research, *auxiliary nurses* refer to individuals employed by a mental healthcare institution to render basic nursing care to patients with intellectual disabilities.

### Research design

A qualitative descriptive inquiry was conducted, as described by Botma et al. (2010:194) and Sandelowski (2000:335). This was deemed an appropriate design for this study, as the purpose of this design was to explore and describe phenomena as they naturally occur. In this case, the perceptions of auxiliary nurses caring for patients with intellectual disabilities on their resilience were explored together with the protective and vulnerability factors that play a role in their resilience when providing nursing care to these patients.

## Research method

### Population and sampling

The study population comprises 220 auxiliary nurses providing nursing care to patients with intellectual disabilities working in a South African mental healthcare institution. The researcher made use of purposive sampling to select the participants. Inclusion and exclusion criteria were used to select the sample for this research study (Botma et al. 2010:200). The researcher selected auxiliary nurses who met the following inclusion criteria: They must be employed at a mental healthcare institution that offers long-term inpatient care for individuals with intellectual disabilities, they must be enrolled at the South African Nursing Council as auxiliary nurses and they must be able to communicate in English or Setswana. The sample selection included auxiliary nurses who have worked a minimum of 6 months and excluded newly employed auxiliary nurses who have worked less than 6 months in providing nursing care to patients with intellectual disabilities.

The sample size comprised 32 auxiliary nurses, namely eight per focus group, and was determined by data saturation after the completion of four semi-structured focus group interviews. The researcher and the supervisor reflected on the first focus group interview before data collection continued to ensure that the participants understood the interview questions and to clarify whether any changes need to be made. No changes were needed.

#### Data collection method

The researcher used semi-structured focus group interviews with a minimum of four to eight participants per group (Botma et al. 2010:211). The researcher used the dynamics of each group to gain information about specific issues − the interactions taking place within groups can highlight and provide rich data on a specific phenomenon (Doody, Slevin & Taggart 2012:1). A semi-structured format with clear open-ended questions was used during the interviews (Krueger & Casey 2014:7). The researcher asked the participants the following questions:

- What do you think is your resilience when providing nursing care for patients with intellectual disabilities?
- What do you see as your protective mechanisms and vulnerability factors that play a role in your resilience when providing care for patients with intellectual disabilities?

Four semi-structured focus group interviews were conducted at a public mental healthcare institution. The first focus group interview was conducted on 21 September 2015, the second focus group interview was conducted on 13 October 2015, the third focus group interview was conducted on 27 October 2015 and the last focus group interview was conducted on 30 October 2015.

#### Data analysis

An inductive content analysis was applied in this research study (Forman & Damschroder 2008:40). In qualitative descriptive studies such as this study, a process of content analysis involves generating codes from data during the course of a study and applying these codes systematically (Sandelowski 2000:338).

In the first step of data analysis, the researcher became immersed in the data trying to obtain a sense of the interviews and wrote memos that assisted him in obtaining a sense of the data (Creswell 2013:183). In the second step, which is referred to as the reduction phase, the researcher developed a systematic approach to reduce the amount of data to relevant data by answering the research questions (Forman & Damschroder 2008:48). A final step in a data analysis involved the explanation of data or when meaning is attached to data (Creswell 2009:186). During this phase of an analysis, codes were put together to improve an understanding of the data (Forman & Damschroder 2008:56). It is a process that includes the formation of themes from codes, and then, these themes were organised into larger units of abstraction to make sense of the data (Creswell 2013:187).

#### Measures to ensure rigour

To ensure trustworthiness, the researcher applied the four suggested criteria outlined by Lincoln and Guba (cited by Botma et al. 2010:234; Krefting 1991:215–222; Polit & Beck 2014:323). To ensure truth value, the researcher used the strategy of prolonged engagement by engaging himself with the participants and the data for a prolonged time. To ensure consistency, the researcher comprehensively described the whole research process, including the exact methods used when the data gathering and the data analysis took place. To ensure neutrality, the researcher used the strategy of an inquiry audit by involving a co-coder for an independent analysis. To ensure applicability, the researcher used purposive sampling to select participants to maximise the range of specific data obtained about the research topic.

#### Ethical considerations

Ethical clearance was obtained for this study (Reference number NWU-00043-15-A1). The researcher further ensured that the research was conducted in an ethical manner by applying ethical principles as prescribed by the Declaration of Helsinki (Brink, Van Der Walt & Van Rensburg 2012:33), that is, three fundamental principles that guide researchers: respect for persons, beneficence and justice.

#### Findings

Five main themes emerged that describe this common understanding. These findings are discussed and supported by direct quotations from the semi-structured focus group interviews.

**Theme 1: Practical wisdom:** The participants revealed that exhibiting *a positive mindset* at work is a form of practical wisdom that keeps them motivated to go on. Their positive mindset is characterised by feelings of hope towards the future. The participants revealed their positive mindset by mentioning that they want to leave a legacy when working with these patients. It was evident that the participants use their conscious knowledge of their own feelings and desires as practical wisdom to build their resilience when providing nursing care to patients with intellectual disabilities. This *self-awareness* helps them to be aware of the fact that they should not return aggressive behaviour when patients display aggressive and challenging behaviour.

The participants furthermore mentioned that their practical wisdom of *assertiveness* − being able to express themselves effectively and standing up for their point of view − helps them to remain resilient when providing nursing care to patients with intellectual disabilities. The participants felt that this firm approach sends a clear message to patients and they never waver from decisions taken. The participants identified that *passion for their work* helps them to remain resilient when providing nursing care to patients with intellectual disabilities. Their intense love for their profession made them realise the reasons they are employed − to render care to patients with intellectual disabilities:

‘*Se se ntirang strong … Ke batla go achive se seng se e leng goreng mo nakong e e tlang at least go bonagale gore ke berekile ka patient.* (What makes me strong is that I want to achieve something that in future it can be noticed that I worked with the patient.)’ (FG 2, P1, male)

‘No is no. If the patient does a wrong thing and I realise that he is doing it intentionally, I confront him.’ (FG 2, P1, female)

‘*I think ke tshwanetse ke be le ho rata mosebetsi wa teng. Ha ke rata mosebetsi, ke kgona go tswelelapele although dipatient di re etsa metlholo 1, 2, 3 e re leng mo go yone.* (I think must love my work. Because if I love my job I can continue although the patients continue to do those things to us.)’ (FG 3, P3, male)

**Theme 2: Interactions used by the participants:** From their responses, it was evident that the participants prefer and enjoy *parent–child relationships* with their patients. They revealed that they prefer to treat patients with intellectual disabilities the same way they treat their own children. The participants mentioned that when they remain *calm and polite* during conflict situations, they are able to manage aggressive and challenging behaviour of their patients effectively. The participants highlighted that effective nurse–patient communication, proper interaction and good relationships are important factors that have a significant impact on the quality of nursing care rendered to these patients.

The participants maintain an appropriate *physical distance and maintain clear boundaries* when interacting with patients with intellectual disabilities. The participants indicated ‘a physical distance’ as an appropriate therapeutic measure that assists nurses when patients are physically aggressive. At the same time, participants revealed their feelings of *sympathy and empathy* as therapeutic factors when providing nursing care to patients with intellectual disabilities. They acknowledged the importance of putting themselves in the shoes of other persons – to have an understanding of the condition or situation of patients with intellectual disabilities.

The participants indicated that they *provide informal rewards* as a method of interaction and agreed that they rewarded the patients with anything available, such as food, to encourage them to change their behaviour and to behave appropriately. The following quotations support the above-mentioned findings:

‘*Obviously o ya go mo treata jaaka ngwana wa gago.* (So obviously you are going to treat him like your child.)’ (FG 1, P1, male)

‘You must be calm and talk to that patient in a polite manner, and you will see later that he will withdraw.’ (FG 2, P1, male)

‘*Ke tshwanetse ke emele kgakalanyana ke tla re motlhomongwe ka moragonyana ka kgona ke buisana le ene ke le mo gaufi.* (I must stay far away. I will maybe come to him later to talk to him while nearer.)’ (FG 3, P3, male)

‘*Nna ke ba kryela jaarmer. Naa bothata ba aka Ke kgona ho mo mamella, ke phole.le ha aka lwana, ha kemo rasetse. Ke a phola.* (I feel sorry for them. My problem is that I can be resilient and relax, even if he can fight. I don’t shout at him. I just calm down.)’ (FG 2, P4, female)

‘You can give him sweets, cigarette, or anything that can make him calm.’ (FG 3, P4, female)

**Theme 3: Strategies used by the participants to remain resilient:** The participants revealed that when they *utilised the induction* given to them, it helps them to be strong and they can continue to render care to patients with intellectual disabilities. They also highlighted the importance of inductions rendered informally by nurses who are working with these patients on a long-term basis. The participants were of the opinion that they get to know the patients more easily when they receive information from experienced nurses. The participants also mentioned that they empower themselves by a *willingness to learn* – they need to understand the patients. By studying the files of patients, they obtain valuable insight about the patients they are providing nursing care to, their level of intellectual disability and the reasons for why they are behaving in a certain way:

‘… if you are new, you will start to be afraid of them, because you don’t know them and begin to think that they are rough and might be danger to me.’ (FG 3, P2, female)

‘We read their files and check how much is the patient scored regarding the level of intellectual disability.’ (FG 1, P1, female)

**Theme 4: The perceptions of the participants on protective mechanisms:** The participants firstly mentioned *trust in God* as a protective mechanism. According to them, God is their protector who they rely on. They added that God always gives them strength to remain strong despite the difficulties they encounter when providing nursing care to patients with intellectual disabilities. The participants revealed that it is important to all the staff members to pray together in the morning before they start with their daily routine. The participants emphasised the importance of *knowing and loving their patients*. They mentioned that it helps them to respond effectively to the needs of their patients. They added that when they know their patients, their understanding makes it easier to respond appropriately, especially during situations when patients display aggressive behaviour. Moreover, the participants highlighted the importance of working in harmony with their peers. They view *positive working relationships* with peers as a strength that empowers them to bounce back despite adversities and to continue rendering nursing care to patients with intellectual disabilities. Another protective mechanism that they use when providing nursing care to patients with intellectual disabilities is by implementing disciplinary measures to assist them in controlling patients. The participants stated that they *discipline* patients who display unruly behaviour.

The above-mentioned findings are supported by the following quotations:

‘*… ya thapelo e a mperekela and e mperekela ka metlha le matsatsi ka gore ha ke tswa ke a rapela, and then ke a kopa ko modimong.* (Of prayer because I saw that it works for me. It works for me forever because when I leave I pray. I make a plea from God.)’ (FG 3, P4, female)

‘*Jaaka balwetsi ba rona ba le difficult, o tlamehile wena o itse patient. O itse behaviour ya patient. And then behaviour ya patient, o tlamehile o itse gore o mo calm down how.* (As our patients are difficult, you are supposed to know your patient. You must know his behaviour and know how to calm him down.)’ (FG 3, P2, female)

‘*Ha o sebetsa le colleague ya haho, le tshwanetse le be team e one, gore le kgone go thusana mo bothata bo leng teng.*’ (When you work with your colleague you must be a one team, so that you can help each where there is a problem.) (FG 4, P7, male)

‘*… patient ha e entse hosa, discipline. rona yaanong re ba solve ka hore tje ka a entse bothata, o mo tima dijo. A ka setlhole a e entse hape ntho eo.* (If the patient has done something wrong, I discipline him. We solve him by not giving him his food. After that, he won’t repeat the same mistake.)’ (FG 2, P4, female)

**Theme 5: The perceptions of the participants on vulnerability factors:** The participants mentioned that they are unable to handle situations effectively when poor communication is used, usually caused by *a lack of appreciation* and when patients do not listen properly. These situations cause the participants to lose focus and their resilience is weakened. The participants also revealed that sometimes they are impatient with their patients because of work-related factors. They mentioned that *impatience* puts them in a vulnerable position and can result in impulsive decisions:

‘*Nako e ngwe ga ba re appreciate. E go senyetsa letsatsi.* (Sometimes they don’t appreciate us. It makes your day a hell.)’ (FG 3, P2, female)

‘*Nako e ngwe kena le go felelwa ke pelo. Sometimes e le gore patient e go kgotle kgakala.* (Sometimes I lose temper. There are times when patient irritate you too much.)’ (FG 2, P6, female)

## Discussion of findings

From the data collected, the auxiliary nurses perceived resilience as the application of their practical wisdom (a positive mindset, self-awareness, assertiveness and passion) when providing nursing care to patients who are intellectually disabled. Literature confirms that practical wisdom refers to the tendency of being morally skilful when challenges are anticipated (Beabout 2012:426). Practical wisdom includes aspects, such as loyalty, self-control, courage, generosity, gentleness, motivation and kindness, which all form part of resilience (Aldwin & Igarashi 2012). With a positive mindset, self-awareness, a conscious knowledge of their own emotions and reactions towards patients can be applied to manage themselves and to enhance their resiliency.

The auxiliary nurses also make use of different forms of interactions to provide nursing care to patients with intellectual disabilities: a parent–child relationship, calmness and politeness, maintaining a safe physical distance and clear boundaries, having sympathy and empathy, and providing informal rewards. In a study that investigated the characteristics of mental health staff interactions with their patients, Gildberg, Elverdam and Hounsgaard (2010:361) also found that nurses tend to regard patients with intellectual disabilities as persons who are not aware of their own behaviour and not able to follow rules and that is why nurses focus on the behaviour of patients as ‘parents’.

The auxiliary nurses prefer calm and polite interactions among themselves and their patients to foster a harmonious atmosphere and to develop quality therapeutic relationships − even by offering patients informal rewards. A similar study also reported that other measures, such as rewarding patients with coffee or access to the television, are used to change the behaviour of patients (Nash & Romanos 2010:686). With regard to calmness and politeness, literature confirms that conflict situations can be handled more effectively when nurses establish harmony by behaving calmly and politely (Konishi et al. 2009:632).

Maintaining a safe physical distance and clear boundaries is one of the measures that the auxiliary nurses use in order to remain resilient. They try to maintain harmony by staying alert, keeping an appropriate physical distance between themselves and patients, and maintaining clear boundaries. Although an appropriate physical distance is kept, the auxiliary nurses continue to interact with their patients by offering them support when needed. Support is usually characterised by feeling empathy even if their patients are challenging. Literature highlights that nurses are able to apply strategies of compromise when patients are difficult − relationships are not possible without empathy (Michaelsen 2012:96).

The auxiliary nurses perceive inductions and their willingness to learn as helpful strategies to remain resilient. They utilise informal inductions received from their experienced peers to get to know their patients better. Inductions improve their self-confidence and resiliency. Their willingness to learn about intellectual disabilities helps them with providing nursing care to their patients, and ultimately, they are able to rise above their daily challenges despite the adversities experienced at their workplace. Literature supports this finding − nurses with a high resilience use positive coping skills, such as identifying resilient role models to learn from and to enable them to continue working in a stressful environment (Mealer et al. 2012:1449).

It was evident from the perceptions of the auxiliary nurses providing nursing care to patients with intellectual disabilities that protective mechanisms play an important role in their resilience. They perceive their trust in God, knowing and loving their patients, positive peer working relationships and applying disciplinary measures on patients as part of protective mechanisms that play a crucial role in their resiliency.

Altaf and Awan (2011:99) mention that nurses have a need to be provided with workplace spirituality to help them love their job and to continue working without quitting. Bundgaard et al. (2012:2287) are of the opinion that knowing patients is important, because knowledge helps nurses to restrict their efforts to the needs of patients and allows them to render care to patients as unique individuals. However; it is evident from the findings that not all of these protective mechanisms are effective − disciplining patients may not be constructive at all.

The auxiliary nurses are of the opinion that vulnerability factors, such as a lack in appreciation and impatience, play a role in their resilience. Work-related factors play a role, such as poor communication, a high work load and a shortage in staff. In a study conducted to explore the lived experiences of nurses who care for mental healthcare users, the nurses felt unappreciated by their supervisors (Sobekwa & Arunachallam 2015:7). This issue of not being given the necessary support by supervisors was also mentioned by participants in a study that identified − from the perspective of nurses − occupational stressors and ways in which these stressors can be reduced (Happell et al. 2013:641).

## Limitations of the research

Although the researcher planned to conduct the semi-structured focus group interviews in English (an inclusion criterion), he allowed the participants to communicate in their own language on their request. This can be viewed as a limitation, because some prospective participants were excluded who actually could have participated. Nevertheless, data saturation was reached. Also, there was a risk that the meaning of discussions was lost in translation. A small sample was used in the research study, because the study was conducted with the aid of participants from a single mental healthcare institution. Some of the participants were outspoken and others were shy. These limitations were addressed to a great extent through an accurate translation and using effective communication techniques during the focus group interviews.

## Recommendations

### Nursing practice

Aspects that threaten the resilience of auxiliary nurses (not being appreciated and being impatient) should be given urgent attention by nursing managers and supervisors. Fostering resilience in workplaces through the introduction of informal peer support among auxiliary nurses is recommended − especially to novice auxiliary nurses. This study identified ethical implications that need to be addressed. This pertains to the application of disciplining measures to modify the behaviour of patients with intellectual disabilities.

Appropriate nurse–patient communication should be improved and can have a significant impact on the quality of nursing care.

### Nursing education

Nursing educators should review the curricula concerning the training of auxiliary nurses. A revision of curricula should include teaching student auxiliary nurses the fundamentals and basics of psychiatric nursing. Knowledge of resilient behaviour has the potential to enhance the clinical repertoire of auxiliary nurses. Nursing education institutions should, therefore, consider including resilience training to equip their students to be able to handle adverse working conditions when providing nursing care to patients with intellectual disabilities. Continual education and skills development at workplaces should be encouraged to allow auxiliary nurses to use their practical wisdom to care for patients with intellectual disabilities.

### Nursing research

This research was conducted in only one mental healthcare institution, and it is recommended that research should be conducted on a larger scale to improve the quality of care rendered to patients with intellectual disabilities. Furthermore, nursing researchers pursuing studies focusing on resilience should identify how the strengthening of the practical wisdom of nurses, interactions with patients with intellectual disabilities, and strategies used by auxiliary nurses affect the resilience of nurses.

## Conclusion

Having to deal with aggressive patients while not trained to do so was experienced by the auxiliary nurses as an adversity and they need to develop resiliency. The outcome of the study indicated that the auxiliary nurses need to be resilient. Resilience can be achieved by applying their practical wisdom, by interacting with patients with intellectual disabilities and by using their own strategies and effective protective mechanisms.

Although the auxiliary nurses did not emphasise having to deal with aggressive patients as an adversity in their workplace, they endure vulnerability factors on a continual basis when providing nursing care to these patients.
